# Neck Loads During Head-First Entries into Trampoline Dismount Foam Pits: Considerations for Trampoline Park Safety

**DOI:** 10.1007/s10439-022-02945-w

**Published:** 2022-04-05

**Authors:** Tom Whyte, Edward Lind, Adam Richards, David Eager, Lynne E. Bilston, Julie Brown

**Affiliations:** 1 Neuroscience Research Australia, Margarete Ainsworth Building, 139 Barker St, Randwick, NSW 2031 Australia; 2 School of Medical Sciences, Faculty of Medicine, The University of New South Wales, Sydney, NSW 2052 Australia; 3 The George Institute for Global Health, Level 5/1 King St, Newtown, NSW 2042 Australia; 4 School of Mechanical and Mechatronic Engineering, Faculty of Engineering and Information Technology, University of Technology Sydney, 15 Broadway, Ultimo, NSW 2007 Australia; 5 Mr Trampoline Pty Ltd, 966-968 & 972 Dandenong Road, Carnegie, VIC 3163 Australia; 6 Prince of Wales Clinical School, Faculty of Medicine, The University of New South Wales, Sydney, NSW 2052 Australia

**Keywords:** Foam pit, Dismount pit, Trampoline park, Cervical spine injury, Head-first impact, Biomechanical testing

## Abstract

Serious cervical spine injuries have been documented from falls into foam pits at trampoline parks. To address the lack of evidence on how foam pits should be designed for mitigating neck injury risk, this study aimed to quantify neck loads during head-first entry into varying foam pit designs. An instrumented Hybrid III anthropomorphic test device was dropped head-first from a height of up to 1.5 m into three differently constructed foam pits, each using a different mechanism to prevent direct contact between the falling person and the floor (foam slab, trampoline or net bed). Measured neck loads were compared to published injury reference values. In the simplest, foam-only pit design, increasing foam depth tended to reduce peak compressive force. At least one injury assessment reference metric was exceeded in all pit conditions tested for 1.5 m falls, most commonly the time-dependent neck compression criterion. The results highlight the importance of adequate foam depth in combination with appropriate pit design in minimizing injury risk. The risk of cervical spine injury may not be reduced sufficiently with current foam pit designs.

## Introduction

Trampoline use is a popular recreation activity and a proposed therapy with many benefits associated with exercise and socializing.^6,23^ However, there is a long recognized risk of serious injury using trampolines domestically.^4^ Trampolines have recently moved into the commercial market as trampoline parks. Trampoline parks typically consist of connected trampolines and often include dismount devices such as foam pits into which participants can jump. An American Academy of Pediatrics policy statement from 2012, reaffirmed in 2020, states that the effect of trampoline park facilities on trampoline injury trends is not yet evident.^4^ A study from New Zealand found trampoline-related injury appears more prevalent with the emergence of trampoline parks, finding a 7-fold increase in trampoline-related hospital presentations after the opening of two parks.^17^ In Australia and Canada, trampoline parks are unregulated, prompting recent calls for better regulation of these facilities.^13,22^ In the absence of regulations, a number of voluntary standards and codes of practice promote minimum safety standards at trampoline parks.^1,2,13^

Foam pits have origins in gymnastics training, used by athletes practicing movements where there is potential for landing dangerously.^8^ They are contained spaces filled with loose foam that reduce acceleration experienced by the body upon landing,^19^ or a combination style dismount pit designed with a rebound device, covered with loose impact absorbing blocks, as referred to in a standard for trampoline courts.^1^ However, injuries occur during falls into foam pits, including serious cervical spinal cord injuries.^9–11^ Published case studies have documented bilateral facet dislocation with spinal cord injury sustained by a gymnast somersaulting into a 1.52 m deep foam pit and a trampoline park patron diving into a 0.65 m deep pit.^10,11^ The nature of the bottom of these foam pits was not described in either case. Suggested mechanisms for foam pit injuries include penetration of the loose foam and striking the bottom of the pit or the foam becoming firmer if not regularly ‘fluffed’,^18^ however the behavior of the spine in head-first impacts into loose foam pieces such as in a foam pit has not been studied.

The risk of injury from diving into foam pits is known, with voluntary standards including signage and/or rules prohibiting diving into foam pits due to this risk.^1,2^ However, the risk may not be known worldwide. A documented complete spinal cord injury case in South Korea reports that the incident occurred from diving into a foam pit designated for activities such as jumping and diving,^10^ suggesting diving was not prohibited. Furthermore, head-first entries into foam pits can occur unintentionally. This study was motivated by understanding how neck injury risk could be minimized in these circumstances.

Bilateral facet dislocation of the cervical spine involves the superior vertebral body being displaced anteriorly over the adjacent inferior vertebra, dislocating both facet joints.^11^ Controlled biomechanical experiments have demonstrated compression and compressive buckling of the cervical spine as the primary cause of this injury.^16^ In head-first impacts, catastrophic cervical spine injuries occur when the head stops and the momentum of the following torso compresses the neck between the torso and head with enough energy to cause injury.^16^ The cervical spine is capable of managing impacts equivalent to a head-first vertical drop of only 0.5 m.^12^ While padded materials are often used effectively in injury prevention applications to absorb energy and reduce peak force and acceleration,^21^ a padded impact surface may increase the risk of cervical spine injury during a head-first impact by delaying and diminishing head motion that might allow the neck to escape compression from the following torso, even though the padding might successfully lower accelerations experienced by the head.^3,11^

The Hybrid III 50th percentile male anthropomorphic test device (ATD) has been used to quantify neck loads in head-first impacts occurring in rollover vehicle crashes and American football. Experiments reproducing cadaver impacts using the ATD have shown that the onset of axial load is similar with the ATD and cadaveric specimens but the axial load response diverges when human specimens fail.^24^ Higher peak ATD loads correlated with cadaver experiments that had a high incidence of injury nonetheless,^24^ and injury assessment reference values have been developed for axial compression of the Hybrid III neck.^14,24^

Typical jump heights at trampoline parks are likely between 0.75 m measured for standing jumpers of varying skill level and 2.5 m achieved by experienced trampoline athletes on a competitive trampoline.^5,20^ Falling into a foam pit from these heights requires considerable energy to be managed by the neck in a head-first entry. Suggestions for ensuring foam pit safety include installing a foam slab or trampoline/net bed beneath loose foam cubes as a fail safety system,^2^ but these have yet to be examined experimentally to our knowledge. Specifications for foam pit designs are included in voluntary standards and codes of practice used by trampoline park operators,^1,2^ but we are unaware of any peer reviewed literature on foam pit injury risk that informed these specifications, nor is any research referenced in these specifications. The cervical injury mitigation value of foam pits constructed to meet these specifications remains unknown. The aim of this study was to quantify neck loads during head-first entries into a range of foam pit designs to examine the cervical spine injury risk.

## Materials and Methods

Neck loads in head-first falls into foam pits were examined by dropping an instrumented Hybrid III 50th percentile male ATD head-first from a variety of drop heights up to 1.5 m, into a range of differently constructed foam pits.

### Drop Testing Setup

**Figure 1:**
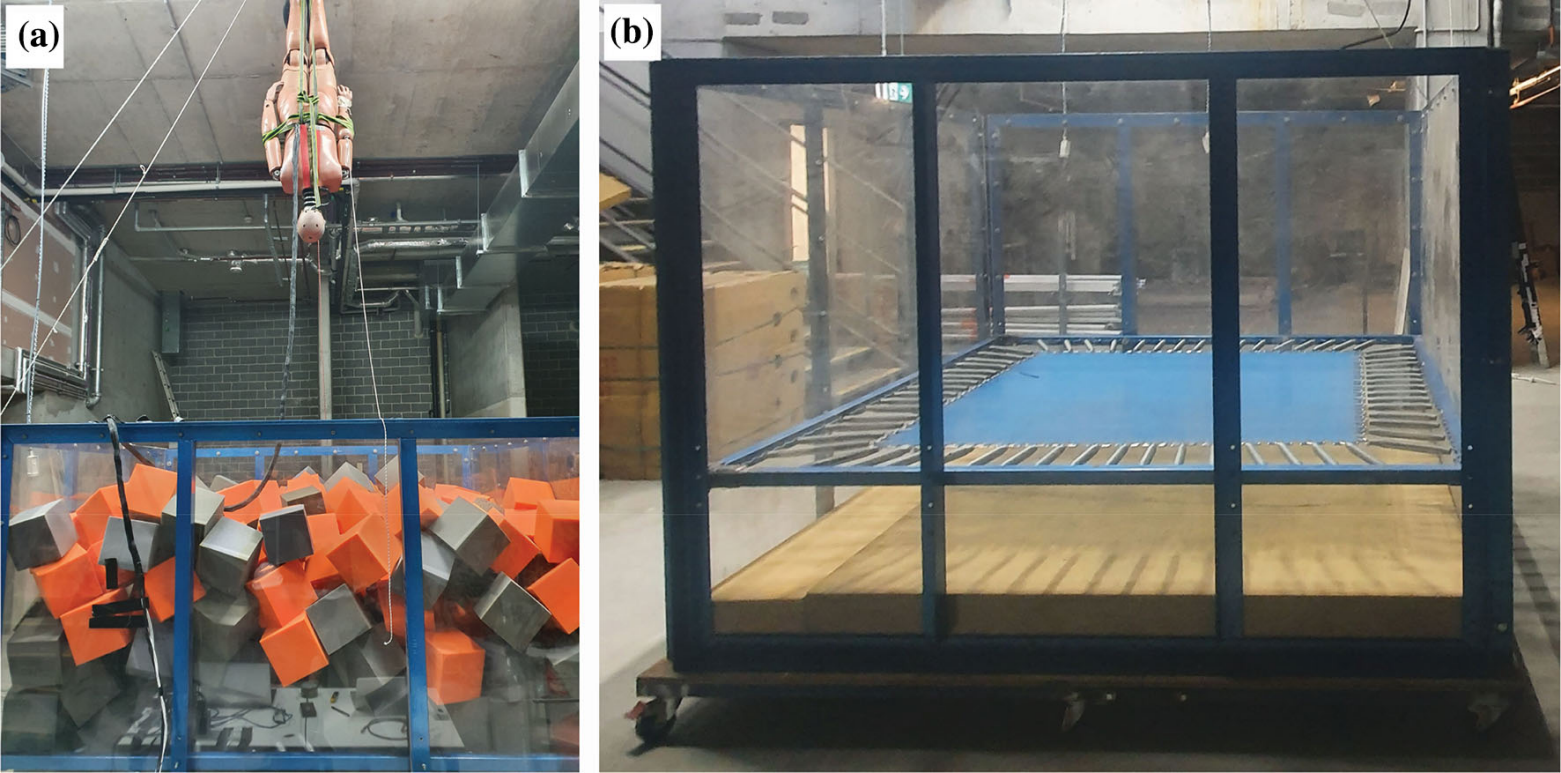
(a) Inverted Hybrid III ATD prior to drop test into filled pit; and (b) custom foam pit with trampoline bed and 0.2 m foam slab at the base without foam cubes.

The ATD was inverted with webbing secured around the ankles. The webbing was attached to a quick release mechanism and then to a rope and pulley system through a fixture in the laboratory ceiling. The ATD was positioned so that the orientation of the body was approximately vertical, the neck was approximately 6 degrees from vertical (slightly flexed compared to a straight body and neck) and the base of the head was horizontal. The arms of the ATD were secured to the sides of the body, consistent with the arm positions of a video-documented case of bilateral facet dislocation from diving into a trampoline park foam pit.^10^ The ATD was released from predetermined heights and allowed to freefall into the foam pit. The setup is depicted in Fig. 1a. Foam cubes were added or removed to achieve an approximately level surface, measured by laying a timber board (2 × 0.076 × 0.012 m^3^) with a levelling device on top. The drop height was the distance between the vertex of the ATD head and the timber board.

The ATD was instrumented with a triaxial array of accelerometers (7231C750T, Endevco, Charlotte, NC) in the head and a 6-axis upper neck load cell (1716A, Denton ATD Inc., Huron, OH). The instrumentation was sampled at 20 kHz with a DTS Slice Pro (Diversified Technical Systems, Inc. Seal Beach, CA), for 2.5 s (0.5 s before time zero and 2 s after time zero) with time zero being when the neck compression force reached 100 N. Accelerometer and force data were filtered according to SAE J211-1. A high-speed camera (Phantom, Vision Research, Wayne, NJ) captured each drop test sampling at 1000 fps. Different aspects of the drop tests were captured by high-speed video in each pit condition and in each test, which included the ATD entering the loose foam for Pits A and B or the deflection of the trampoline/netting for Pit B1 and B2.

### Foam Pit Characteristics

A custom foam pit enclosure was constructed from steel supports, Perspex side panels and a hardwood base overlaying the steel frame, measuring 2.6 m long, 2.6 m wide and 1.9 m high from the base (Fig. 1b).

Foam cubes from a local trampoline park foam pit were used to fill the foam pit enclosure. These cubes had an average side length of 0.2 m (range 0.194–0.205 m) and density of 23.7 kg/m^3^ (range 22.2-25.5 kg/m^3^) determined by measuring ten randomly selected cubes. The manufacturer provided density for the foam was 21.8 kg/m^3^ (method ASTM D3574-08 Test A) and the 25% indentation force deflection (method ASTM D3574 Test B1 for 4-inch-thick sample) rating was 196 N (44 lbs).

The pit was constructed to allow the design of the pit to be varied across three designs based on the ATPA code of practice,^2^ each using a different mechanism to prevent direct contact between the bottom of the foam pit and the falling person, being a foam slab, trampoline or net bed. Due to laboratory space restrictions, the custom foam pit did not meet the minimum length specified for foam pits in either the ATPA code of practice or ASTM F2970-20.

#### Pit A: Foam Slab Only

**Figure 2:**
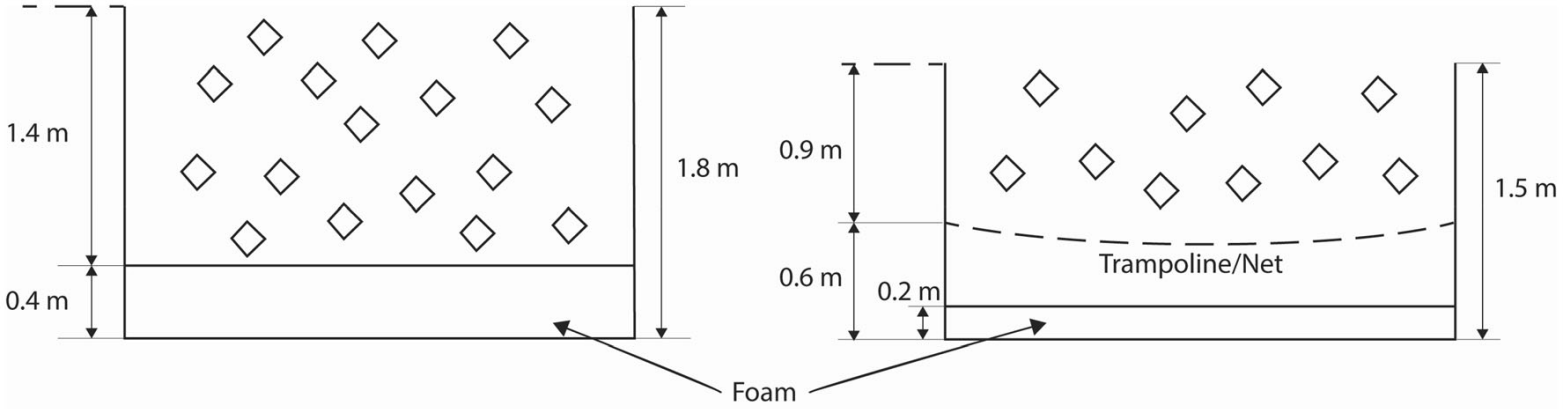
Dimensions of Pit A (left) and Pit B (right) showing foam cubes and foam slab with shading. The trampoline or netting bed is shown partially deflected with a dashed line.

Pit A consisted of a foam slab 0.4 m thick at the base of the Foam cubes filled the pit to a depth of 1.4 m above the foam slab, or to a depth of 1.8 m from the base of the pit (Fig. 2).

#### Pit B1: Trampoline Bed and Foam Slab

Pit B1 consisted of a foam slab 0.2 m thick at the base of the pit. A trampoline bed was mounted onto horizontal steel supports 0.6 m from the base of the pit (see Fig. 1b). Foam cubes filled the pit to a depth of 0.9 m above the trampoline bed, or to a height of 1.5 m from the base of the pit (Fig. 2). Pit B1 was similar to the trampoline court foam pit design specified by ASTM F2970-20,^1^ but differed slightly in depth dimensions (F2970-20 requires a minimum of 60 inches, or 1.524 m).

The trampoline bed was a woven and painted 2-string performance bed (MrTrampoline, Carnegie, VIC, Australia). The bed material was a polyester terephthalate yarn with 16 threads per inch, 8 ends per inch (warp) and 8 picks per inch (weft), coated in a chlorinated rubber paint. The bed was attached to the foam pit frame with 62 performance springs each 230 mm in length prior to elongation. These springs stretched 10 mm when a mass of 9.33 kg was attached to one end.

#### Pit B2: Netting Bed and Foam Slab

Pit B2 was the same as Pit B1 except instead of a trampoline bed, tightly fitting netting was fastened to the horizontal steel supports 0.6 m from the base of the pit using cable ties (Fig. 2).

The netting was black 2 mm diameter 60 ply Nylon with rope edging and square netting gaps sized 40 × 40 mm. The size of this net was made to the measurements within the steel supports of the foam pit (236 × 239 mm) (MrTrampoline, Carnegie, VIC, Australia).

### Test Conditions

As shown in the test matrix (Table 1), drop heights ranged from 0.1 to 1.55 m and varied across the different foam pit designs. Depth of foam cubes was also varied from 0 to 1.4 m to examine the ATD neck compression response from directly contacting the fail safety system of each pit configuration, and in the specified design conditions.

**Table 1:** Test matrix.

Pit	Depth of foam cubes (m)	Base foam thickness (m)	ATD drop height (m)	Trampoline drop location	Number of drops	Test ID numbers (TFPXX)
A	0	0.20	0.10	Centre	1	01
A	0	0.20	0.25	Centre	1	02
A	0.45	0.20	1.15	Centre	1	21
A	1.00	0.40	1.15	Centre	2	17–18
A	1.40	0.40	1.15	Centre	3	14–16
A	1.00	0.40	1.55	Centre	2	19–20
B1	0	0.20	0.35	Centre	1	03
B1	0	0.20	0.61	Centre	1	04
B1	0	0.20	0.60	Offset	4	25–28
B1	0.90	0.20	1.00	Centre	1	05
B1	0.90	0.20	1.50	Centre	8	06–13
B1	0.90	0.20	1.50	Offset	3	29–31
B2	0	0.20	0.60	Offset	1	32
B2	0.90	0.20	1.50	Offset	3	33–35

For Pit A, a drop height of 1.5 m could not be achieved at the full foam pit depth of 1.8 m due to ceiling height restrictions. Drop tests were instead performed at the maximum achievable 1.15 m into the full foam pit depth of 1.8 m as well as drop tests from 1.55 m into a reduced foam pit depth totaling 1.4 m (0.4 m foam slab and 1.0 m cubes). A drop test was also performed with a total foam depth of 0.65 m (0.2 m slab and 0.45 m cubes), matching the pit depth of a documented bilateral facet dislocation case.^10^ This drop test was performed from a height of 1.15 m above the foam cubes, a height estimated to be reached by the jumper in video documentation of the incident.^10^ All drops into Pit A were centered in the middle of the pit.

The 1.5 m drop height could be achieved with Pit B1. Drop tests into Pit B1 were performed both in the center and offset from the center of the pit (0.5 m from the center toward one side) since it was thought that the trampoline bed beneath the foam cubes may respond differently depending on the impact location. Very little bed sag due to the weight of the foam cubes was observed for Pit B1.

Two drop test conditions were performed for Pit B2 being the offset location (0.5 m from the center to one side) at two drop heights, see Table 1. The netting bed was observed to sag with the addition of the foam cubes for Pit B2. This bed sag was confirmed to be at least 185 mm above the foam pit floor, in accordance with the ATPA code of practice, but not further quantified.

### Data Analysis and Injury Risk Assessment

Upper neck compression loading rate was calculated during the initial neck compression response and filtered with a 4th order Butterworth low pass filter, 150 Hz cut-off, using a custom MATLAB script (R2018b, Mathworks, Natick. MA).

Flexion-extension bending moments were transformed to the location of the occipital condyles as is standard practice according to CFR Part 572 Subpart E §572.33. Neck injury criterion, *N*_*ij*_, which is a linear combination of axial load and sagittal plane bending moment that can be tolerated by the cervical spine, was calculated using the published out-of-position intercepts (compression and tension 6200 N, flexion moment 305 Nm, extension moment 122 Nm),^15^ chosen as a worst-case scenario in a non-standard procedure. Measured *N*_*ij*_ was compared to the 0.88 value corresponding to 5% probability of unstable cervical spine injury in head-first impact experiments at an initial neck angle of 6° for Hybrid III ATD and cadaver impacts with matched kinematics.^24^

Neck compression force was compared to two further injury criteria developed using the Hybrid III in reconstructed football player impacts.^14^ Firstly, peak axial compression injury reference of 4 kN. Secondly, a time-dependent compressive load threshold above which the neck loading has the potential to produce serious neck injury.^14^ Results are reported for the duration of compression above 1.11 kN which is tolerable for 35 ms according to the time-dependent criterion and the duration of compression above 2.7 kN which is tolerable for 25 ms.

## Results

### Pit A: Foam Slab Base Only

Peak head and neck responses from Pit A testing are shown in Table 2. The drop test reconstructing the 0.65 m pit depth and 1.15 m drop height conditions of a previously reported bilateral facet dislocation case (TFP21, Table 2) resulted in the highest neck compression force (5.74 kN), *N*_*ij*_ (1.76), and longest duration of compressive force greater than 2.7 kN (76 ms). In tests into pits filled with at least 1.0 m of foam cubes, peak neck compression and *N*_*ij*_ were below injury thresholds. In all drop tests the duration of neck compression greater than 1.11 kN was more than 35 ms and in one case (TFP19) the duration of neck compression greater than 2.7 kN was more than 25 ms.

**Table 2:** Pit A, with base foam slab only, peak response test results.

Test ID	Foam cubes depth (m)	Base foam slab thickness (m)	ATD drop height (m)	3 ms resultant head accel. (g)	Posterior shear force Fx (kN)	Anterior shear force Fx (kN)	Lateral shear force |Fy| (kN)	Comp. force Fz (kN)	Comp. force loading rate (kN/s)	Lateral bending moment |Mx| (Nm)	Extension moment Myoc (Nm)	Flexion moment Myoc (Nm)	Axial rotation moment |Mz| (Nm)	N_ij_	Duration Fz < -1.1 kN (ms)	Duration Fz < -2.7 kN (ms)
TFP01	None	0.20	0.10	2.66	− 0.02	0.20	0.17	−1.96	−23.6	11.15	−51.30	1.53	3.91	0.73	**123.0**	0
TFP02	None	0.20	0.25	7.11	− 0.24	0.36	0.16	**−4.62**	−150.6	11.80	−55.08	22.91	1.68	**1.12**	**98.2**	**41.1**
TFP21	0.45	0.20	1.15	16.33	− 1.36	0.35	0.55	**−5.74**	−219.9	38.32	−103.89	30.09	12.01	**1.76**	**133.0**	**76.1**
TFP17	1.00	0.40	1.15	5.67	− 0.36	0.11	0.87	−2.44	−36.0	28.71	−0.60	18.92	27.51	0.43	**126.6**	0
TFP18	1.00	0.40	1.15	7.51	− 1.44	0.12	0.76	−2.22	−48.9	36.12	−5.43	18.63	21.67	0.38	**88.1**	0
TFP14	1.40	0.40	1.15	5.06	− 0.09	0.08	0.76	−1.86	−27.5	48.71	−29.32	8.13	8.47	0.51	**95.3**	0
TFP15	1.40	0.40	1.15	6.10	− 0.55	0.14	1.09	−1.57	−24.8	20.71	−3.22	48.71	36.44	0.34	**78.2**	0
TFP16	1.40	0.40	1.15	5.22	− 0.10	0.63	0.35	−1.94	−25.9	45.23	−30.59	7.09	25.10	0.46	**110.5**	0
TFP19	1.00	0.40	1.55	7.96	− 1.10	0.13	0.69	−3.11	−61.4	19.89	−39.40	7.42	5.44	0.82	**127.5**	**41.9**
TFP20	1.00	0.40	1.55	8.32	− 0.02	0.68	1.25	−2.68	−54.7	23.87	−63.27	11.75	6.74	0.85	**138.5**	0

Values exceeding injury assessment reference values are bold

**Figure 3:**
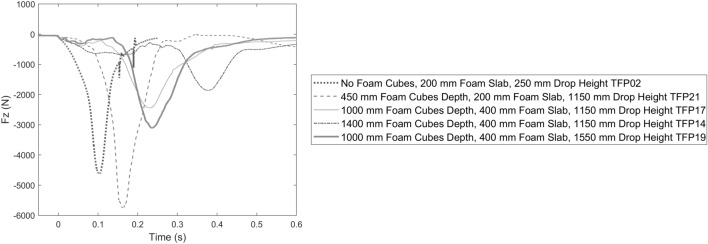
Neck compression force Fz over time for Pit A tests with varying depths of foam cubes, foam slab thickness and ATD drop heights as labelled.

The temporal neck compression response for varying test conditions in Pit A is pictured in Fig. 3. When foam cubes are absent, there was an immediate rise in compressive force to beyond 4 kN from a modest 0.25 m drop height. Increasing the depth of foam cubes delayed the rise in Fz as well as reduced the peak compressive force when tested at the same 1.15 m drop height. Increasing the drop height into the same foam depth produced higher peak neck compression with a similar time of peak force.

### Pit B1: Trampoline Bed and Foam Slab

Peak head and neck responses from Pit B1 tests are shown in Table 3. In 1.5 m drops into 0.9 m deep foam cubes above the trampoline bed, *N*_*ij*_ ranged from 0.27 to 1.38, with four tests exceeding 0.88. In one of these tests, neck compression exceeded 4 kN. All drops into the center of Pit B1 and one offset from the center produced neck compression forces above 1.11 kN that lasted longer than 35 ms. In three drops into the pit center, neck compression force duration above 2.7 kN was greater than 25 ms. In two of three drop tests from 1.5 m into the 0.9 m filled pit at the offset location, no neck injury criteria were exceeded.

**Table 3:** Pit B1, with trampoline bed and base foam slab, test results.

Test ID	Foam cubes depth (m)	Base foam slab thickness (m)	ATD drop height (m)	Tramp. impact location	3 ms resultant head accel. (g)	Posterior shear force Fx (kN)	Anterior shear force Fx (kN)	Lateral shear force |Fy| (kN)	Comp. force Fz (kN)
TFP03	None	0.20	0.35	Centre	8.92	− 0.10	1.52	0.37	− 2.96
TFP04	None	0.20	0.61	Centre	10.60	− 0.09	1.70	0.33	− 3.62
TFP25	None	0.20	0.60	Offset	8.12	− 0.13	1.57	0.57	− 3.64
TFP26	None	0.20	0.60	Offset	8.12	− 0.47	0.12	0.93	− 3.25
TFP27	None	0.20	0.60	Offset	10.38	− 0.06	1.82	0.44	− 3.67
TFP28	None	0.20	0.60	Offset	9.51	− 0.08	1.61	0.76	− 3.62
TFP05	0.90	0.20	1.00	Centre	5.87	− 1.12	0.01	1.05	− 2.68
TFP06	0.90	0.20	1.50	Centre	10.45	− 1.80	0.56	1.79	− 1.26
TFP07	0.90	0.20	1.50	Centre	13.67	− 0.41	0.47	1.35	**− 5.26**
TFP08	0.90	0.20	1.50	Centre	10.10	− 0.38	0.11	1.47	− 2.62
TFP09	0.90	0.20	1.50	Centre	12.01	− 1.68	0.10	0.28	− 3.34
TFP10	0.90	0.20	1.50	Centre	9.14	− 0.07	1.22	0.54	− 1.45
TFP11	0.90	0.20	1.50	Centre	11.16	− 1.11	0.30	0.66	− 3.24
TFP12	0.90	0.20	1.50	Centre	9.51	− 0.11	0.37	0.89	− 2.65
TFP13	0.90	0.20	1.50	Centre	15.60	− 2.57	0.09	1.07	− 2.02
TFP29	0.90	0.20	1.50	Offset	5.37	− 0.06	0.78	1.22	− 1.06
TFP30	0.90	0.20	1.50	Offset	8.15	− 0.10	1.72	0.75	− 2.02
TFP31	0.90	0.20	1.50	Offset	5.75	− 0.09	0.28	1.15	− 1.01

Test ID	Comp. force loading rate (kN/s)	Lateral bending moment Mx (Nm)	Extension moment Myoc (Nm)	Flexion moment Myoc (Nm)	Axial rotation moment Mz (Nm)	N_ij_	Duration Fz < −1.1 kN (ms)	Duration Fz < −2.7 kN (ms)
TFP03	− 45.1	18.33	− 41.16	66.60	8.40	0.78	**132.3**	**36.7**
TFP04	− 55.4	21.71	− 60.65	86.78	4.67	**1.02**	**122.0**	**57.3**
TFP25	− 50.6	44.60	− 78.91	75.87	5.33	**1.17**	**149.2**	**72.8**
TFP26	− 43.9	30.52	− 83.52	16.44	20.90	**1.05**	**151.5**	**55.2**
TFP27	− 48.4	23.17	− 67.58	59.55	7.79	**1.07**	**128.5**	**61.8**
TFP28	− 51.8	58.22	− 85.82	75.01	22.25	**1.23**	**138.8**	**65.6**
TFP05	− 49.9	37.27	− 15.85	86.49	40.19	0.71	**169.4**	0.0
TFP06	− 24.1	59.65	− 68.71	59.59	96.43	0.74	21.9	0.0
TFP07	− 99.8	100.08	− 63.02	10.26	17.61	**1.14**	**111.3**	**62.8**
TFP08	− 57.1	30.94	− 63.75	13.68	18.63	**0.92**	**83.2**	0.0
TFP09	− 72.9	30.13	− 75.00	5.70	21.26	0.81	**98.6**	**33.6**
TFP10	− 20.2	27.85	− 2.85	28.02	4.27	0.29	**56.2**	0.0
TFP11	− 61.8	43.98	− 105.03	9.17	21.77	**1.38**	**98.5**	**36.1**
TFP12	− 53.4	32.51	− 83.68	11.07	15.09	**1.07**	**97.9**	0.0
TFP13	− 61.4	37.49	− 19.54	105.99	14.87	0.60	**45.6**	0.0
TFP29	− 20.1	105.26	− 12.93	20.63	13.48	0.27	0.0	0.0
TFP30	− 27.0	47.07	− 16.40	54.72	15.17	0.40	**119.2**	0.0
TFP31	− 15.9	41.05	− 51.77	21.98	14.30	0.59	0.0	0.0

Values exceeding injury assessment reference values are shown in bold

**Figure 4:**
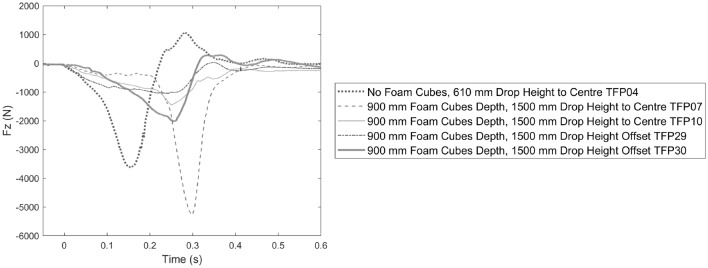
Neck compression force Fz over time for Pit B1 tests with and without 0.9 m depth of foam cubes and at different impact locations on the trampoline bed as labelled.

Neck compression response for head-first drops into Pit B1 is shown in Fig. 4. Time of peak compression was later in 1.5 m drops into 0.9 m deep foam cubes compared to a 0.61 m drop into the trampoline bed and foam slab alone (no foam cubes). In the test without foam cubes, the ATD neck experienced tension upon rebound of the head from the foam slab base (Fig. 4). Drop tests offset from the pit center exhibited a more consistent rise in compression force compared to drop tests in the center which showed an inflection point in the response at approximately 0.22 s.

**Figure 5:**
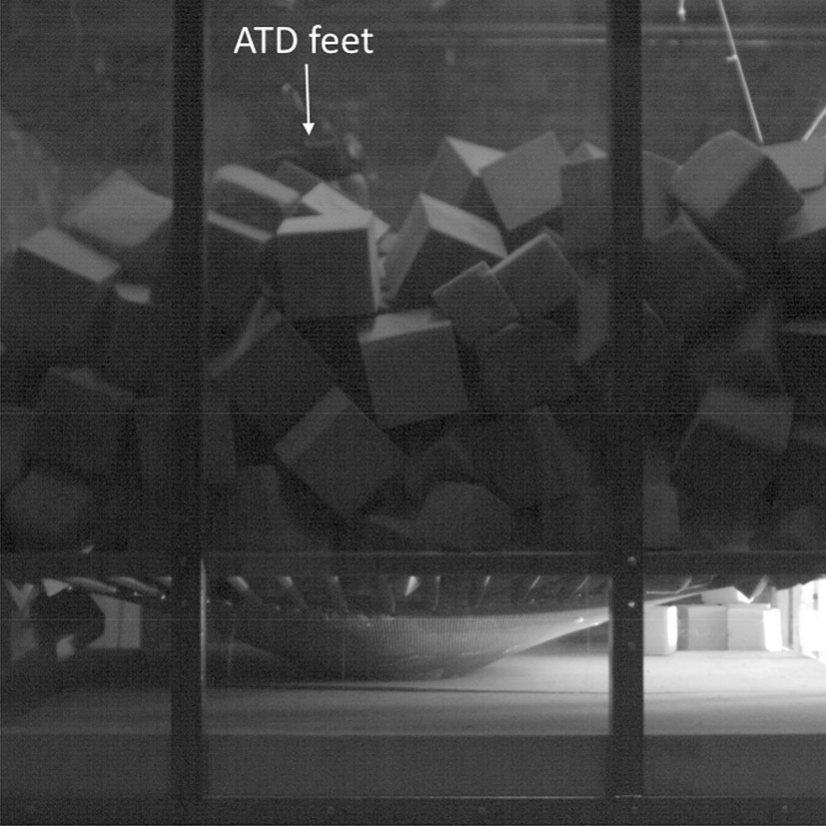
Trampoline bed deflection contacting the foam slab at the base of the pit during head-first entry test ID TFP09. The feet of the ATD are indicated with the white arrow.

The trampoline bed was observed to deflect to contact the foam slab at the base of the pit in all tests. This was viewed in high-speed video specifically positioned to capture the deflection of the trampoline bed for test IDs TFP08, TFP09, TFP10, TFP13 (e.g. Fig. 5).

### Pit B2: Tight Net Bed and Foam Slab

Peak head and neck responses for testing Pit B2 are shown in Table 4. For 1.5 m head-first falls into 0.9 m deep foam cubes above tight netting, *N*_*ij*_ ranged from 0.45 to 1.04 and exceeded 0.88 for one test. None of the three drops exceeded 4 kN of neck compression. Two of the three drops in this condition produced neck compression forces above 1.11 kN with a duration longer than 35 ms while none of these drops had a compression force greater than 2.7 kN. One of three tests did not exceed any neck injury criteria.

**Table 4:** Pit B2, with tight net and base foam slab, test results.

Test ID	Foam cubes depth (m)	Base foam slab thickness (m)	ATD drop height (m)	Net impact location	3 ms resultant head accel. (g)	Posterior shear force Fx (kN)	Anterior shear force Fx (kN)	Lateral shear force |Fy| (kN)	Comp. force Fz (kN)
TFP32	None	0.20	0.60	Offset	12.88	− 0.82	0.39	0.95	**− 5.03**
TFP33	0.90	0.20	1.50	Offset	13.49	− 1.49	0.42	0.26	− 1.62
TFP34	0.90	0.20	1.50	Offset	7.13	− 0.09	0.92	1.04	− 1.59
TFP35	0.90	0.20	1.50	Offset	9.52	− 0.02	0.44	0.51	− 1.02

Test ID	Comp. force loading rate (kN/s)	Lateral bending moment Mx (Nm)	Extension moment Myoc (Nm)	Flexion moment Myoc (Nm)	Axial rotation moment Mz (Nm)	N_ij_	Duration Fz < − 1.1 kN (ms)	Duration Fz < − 2.7 kN (ms)
TFP32	− 107.9	61.18	− 32.26	33.60	11.38	0.83	**123.4**	**81.3**
TFP33	− 33.3	20.48	− 99.43	17.76	10.11	**1.04**	**55.6**	0.0
TFP34	− 21.9	67.71	− 38.46	29.13	19.03	0.54	**74.6**	0.0
TFP35	− 18.8	44.07	− 39.58	8.24	16.84	0.45	0.0	0.0

Values exceeding injury assessment references are shown in bold

**Figure 6:**
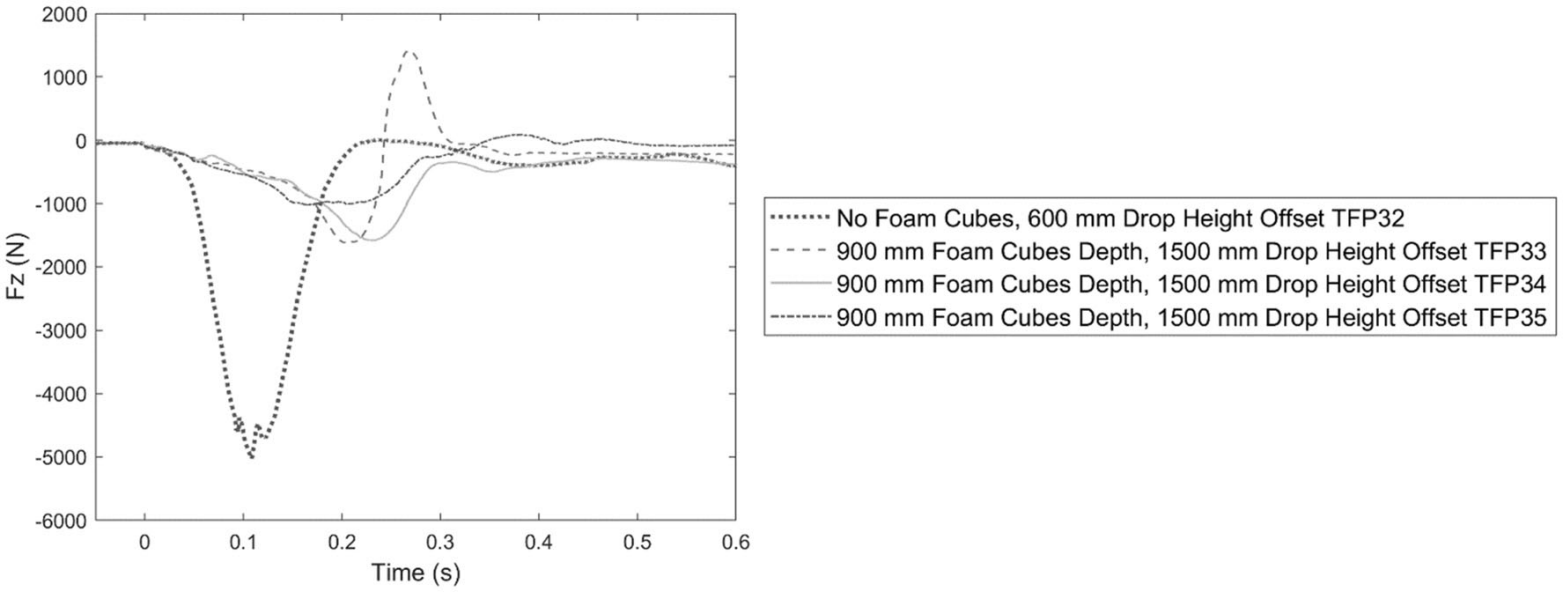
Neck compression force Fz over time for foam Pit B2 tests with and without 0.9 m depth of foam cubes as labelled.

The neck compression response for head-first drops into Pit B2 is shown in Fig. 6. The time of peak compression was later and magnitude lower in 1.5 m drops into 0.9 m deep foam cubes above the netting bed compared to a 0.6 m drop into the netting bed alone above the foam slab.

The netting contacted the foam slab at the base of the foam pit in all three tests with 0.9 m cube depth.

## Discussion

This study is the first to perform biomechanical testing to measure neck loads during head-first entries into foam pits, providing important insights for safety at trampoline parks. The results show that the foam pit designs tested here and based on current specifications can reduce the compressive force acting on the spine in head-first entries. However, the risk of cervical spine injury may not be reduced sufficiently since at least one injury criterion was exceeded in all pit conditions tested for 1.5 m falls of a 50th percentile male (150 cm tall, 78 kg weight). The test results highlight the importance of adequate depth of foam in combination with appropriate pit design in minimizing injury risk.

### Neck Injury Mechanism and Risk in Foam Pit Falls

In most test conditions, at least one published cervical spine injury criteria was exceeded indicating the potential for catastrophic neck injury. Although not a completely faithful reconstruction of a published case report of bilateral facet dislocation from diving into a foam pit,^10^ drop test TFP21 exceeded all three cervical spine injury reference metrics for a fall of similar height into the reported depth of foam.

At the highest foam pit depths, the time-dependent injury criteria was exceeded most regularly, with time durations of neck force above 1.1 kN often much larger (up to 169 ms) than the threshold of 35 ms. The long duration of these compressive forces is not typical of head-first impacts in vehicle collisions or sports. Foam pits allow the head to be slowed over a longer distance than other types of head-first impacts, however the energy of the following torso still needs to be managed by the neck. In previous head-first impact cadaver experiments, compliant padding on the impact surface in some cases increased the risk of neck injury compared to rigid surface impacts, with higher friction between the head and impact surface the primary driver of increased injury risk.^3,11^ Head-first entry into foam pits may be an extreme case of this type of event, however the time-dependent injury reference requires further validation in this context since it was developed reconstructing head-first impacts with loading durations up to 40 ms. The rate of axial load onset when diving into a filled foam pit may differentiate this type of event from other types of head-first impacts.

The complexity of bilateral facet dislocation should be highlighted in interpreting the results. While compression and compressive buckling of the cervical spine is the primary cause of this injury,^16^ other loads such as shear forces, lateral bending and/or axial rotation moments that were not considered here in injury assessment may also contribute. These effects may be exaggerated in diving foam pit entries where a jumper has both horizontal, vertical and rotational velocity, whereas only vertical falls were performed in this study. The tolerance for bilateral facet dislocation may also be lower than injury assessment reference values used here since injury criteria were developed from experiments and reconstructions where the outcome was often vertebral fracture.^14,16,24^

### Foam Pit Response and Test Repeatability

In drop tests into filled foam pits, the neck compression force response generally shows two phases. The first phase begins as the ATD first enters the foam. This provided an initial neck compression response that was not consistent in repeated tests conducted in the same conditions, likely related to a variable amount of loose foam engaged in each impact. The second phase of the neck response occurs when the foam slab at the base of the pit is engaged in the impact, which could be visually confirmed in the case of Pit B1 and B2 and presumed in the case of Pit A based on the estimated penetration depth and the post-test position of the ATD. Phase 2 generally shows an inflection point and sharp increase in the compressive force response (see Figs. 3, 4 and 5). Noting that in all pit designs tested it was not possible for the ATD to contact the pit floor directly, a sharp increase in compressive force likely indicates densification, or “bottoming out”, of the foam slab at the base of the pit. For Pit A, the time duration of phase 1 was related to the depth of foam with no phase 1 in the absence of foam cubes, the shallowest foam cubes (0.45 m) providing the shortest phase 1 (0.1 s) and the deepest pit (1.4 m foam cubes) the longest phase 1 (0.3 s, see Fig. 3), as expected for a greater falling distance from the top of the foam cubes to the foam slab at the base of the pit. For all pit conditions, peak neck compression force occurred in phase 2. Peak response in phase 2 was larger in tests where the upper neck impulse (area under the force-time curve) in phase 1 was smaller, suggesting the amount of loose foam engaged in the initial impact influences the peak compression force. An example of this is the difference between tests TFP07 and TFP10 (Fig. 4), despite being carried out in the same initial conditions. Test TFP07 exhibited a smaller phase 1 impulse compared to TFP10 potentially indicating more loose foam was engaged in TFP10, providing greater cushioning and lower peak neck compression force in phase 2 after a similarly timed inflection point at 0.22 s.

It was not possible to visually assess whether the ATD contacted the foam slab in Pit A. Relying on the observed approximate ATD penetration, post-test position and the biphasic neck compression response, it is most likely that the ATD contacted the slab in all tests. In all Pit B1 and B2 tests, the deformation of the trampoline and netting contacted the underlying foam slab. It was not possible to quantify the observed deformation into the foam slab since the bottom of the trampoline/netting contained foam cubes beneath the falling ATD which did not provide a consistent profile penetrating the slab.

Very little motion or deformation of the loose foam cubes around the edges of the foam pit was observed on the high-speed video in all pit conditions, demonstrating that head-first entries into foam pits are impacts localised to a small region of foam within the pit.

Changes in the fall height and depth of foam cubes in Pit A influenced the neck compression response. An increase in fall height from 1.15 to 1.55 m into the same 1.0 m depth of foam cubes increased the average peak neck compression force (2.33 to 2.90 kN), duration of force above 1.1 kN (107 to 133 ms) and *N*_*ij*_ (0.41 to 0.84). In the tests performed from 1.15 m fall height, the average peak neck compression and the duration of force over 1.1 kN decreased at increased depth of foam cubes. However, *N*_*ij*_ did not follow this trend. An increased depth of foam cubes from 1.0 to 1.4 m for a fall height of 1.15 m resulted in a 23% (0.54 kN) reduction in average peak neck compression force while *N*_*ij*_ increased by 7% (0.03). Drop tests into 1.4 m of foam cubes experienced greater average extension moments at the upper neck (21 Nm) than into 1.0 m of foam cubes (3 Nm) which likely contributed to the rise in *N*_*ij*_. It is not clear whether this difference in flexion-extension bending moment is related to the increased depth of foam cubes or variability in the testing procedure. The relative importance of peak compression force and peak bending moment in contributing to *N*_*ij*_ on injury risk in head-first impact circumstances is also not well-defined. In previous matched Hybrid III and cadaver head-first impact experiments in a similar neck orientation to this study, the contribution of upper neck sagittal plane bending moments to *N*_*ij*_ was an average of 4.1 ± 4.2%.^24^ In contrast, the average contribution of bending moments to *N*_*ij*_ was 38.2 ± 7.2% in the 1.15 m drops into 1.4 m of foam cubes in this study. Nevertheless, both the measured peak compression force and *N*_*ij*_ were below proposed injury assessment reference values while the duration of force above 1.1 kN exceeded the proposed limit. If peak compression force could be reduced to below 1.1 kN, all injury criteria would indicate a low risk of cervical spine injury.

The repeatability of the test procedure varied by test condition and generally showed consistent peak Hybrid III neck compression force in the same test conditions for Pit A (coefficient of variation (CV) in filled pit conditions of up to 11%) and B2 (CV up to 24%) while the consistency for Pit B1, particularly drop tests in the center of the trampoline bed, was poor (CV up to 47%). Variability of ATD peak compression for Pit B1 tests might be explained by the response of the trampoline bed to centrally located impacts. In centrally located impacts, the trampoline springs on each side deflect equally and may allow the trampoline bed to deflect further than in offset impacts where the springs closest to the impact point might reach full extension. In the center impact location, the ATD head is therefore more likely to impact the underlying foam slab at higher velocities, but this is also likely dependent on the variable quantity of loose foam engaged within the foam pit during phase 1 of the neck compression response. Furthermore, it is experimentally difficult to consistently impact the same spot on the trampoline bed due to unavoidable small variations in ATD position pre-impact which inevitably lead to differences in ATD kinematics on impact in the center of the bed.

Trampoline park foam pits are larger in footprint area than the foam pit used for this study meaning the deflection of the springs of a trampoline bed in a real pit is a smaller proportion of the bed area than the custom pit. Hence, the deflection observed in Pit B1 (Fig. 5) likely represents a worst case, whereas a larger foam pit might better prevent impact with the underlying foam. Drop tests in the offset position of Pit B1 provided a more consistent response which may be due to reduced trampoline deflection and potentially more representative of a larger foam pit. Given the custom foam pit size and the variability of ATD responses, further testing is indicated to better understand how pit dimensions contribute to injury risk, particularly for Pit B1.

### Implications for Foam Pit Design and Trampoline Park Standards

The fact that in all test conditions at least one injury reference was exceeded in at least one test suggests that pit depths should be further increased in current specifications if a 1.5 m fall height can be feasibly achieved by a 50^th^ percentile male at trampoline parks.

Pits B1 and B2 appear more effective than Pit A at reducing neck injury risk, with some tests providing ATD responses below all injury references at 1.5 m fall heights. Pit A filled to 1.8 m total depth of foam produced, on average, larger peak compressive neck force from a lower drop height (2.33 kN from 1.15 m drop height) than drops into the offset position of Pits B1 (1.36 kN from 1.5 m drop height) and B2 (1.41 kN from 1.5 m drop height) that have a working depth of 1.5 m. These factors suggest Pits B1 and B2 as preferred designs.

Given the catastrophic consequences of cervical spinal cord injury, administrative controls such as those recommended in ASTM F2970-20 should be used to limit the potential for head-first diving into foam pits as much as possible. Additional research into more effective dismount pit designs is also indicated.

### Limitations

Firstly, the low number of tests carried out for this study limits statistical power needed to make definitive conclusions about aspects of foam pit design. Using the standing Hybrid III allowed the head, torso and legs of the ATD to be aligned and minimized the influence of legs on neck loads observed in previous head-first ATD impact experiments.^7^ However, as discussed, small variations in ATD posture after release were not assessed and could have influenced the neck loads. Using more instrumentation, particularly the lower neck load cell, could provide more insight into better understanding foam pit response in future testing. The tested conditions do not encompass all foreseeable circumstances. The ATD represented a 50th percentile adult male and the pit response will vary for different sized jumpers. A single density of foam cube and slab was used. A string bed trampoline was used for Pit B1, as is commonly used in foam pits in Australia. Using a different type of trampoline material would likely change the response of this pit design. Furthermore, this study did not assess factors related to long term foam pit use such as deterioration of the foam or refluffing of the pit foam cubes which can affect pit response.^18^ Another limitation is the purely vertical fall dynamics for this study compared to someone jumping from an adjacent trampoline where they would have additional components of horizontal and rotational velocity. The response of the head, neck and torso may differ in these simplified fall dynamics compared to a head-first entry from an adjacent trampoline, particularly with respect to constraints imposed on head motion and the potential for escaping the momentum of the following torso. As further testing is required to understand what factors or combinations of factors contribute to injury risk, it is possible that some important but as yet unknown factors may not have been adequately controlled in this set of experiments, particularly for the Pit B1 design. Finally, the injury references represent the current best knowledge for assessing head-first impact injury risk with the Hybrid III ATD but their accuracy and sensitivity in foam pit conditions cannot be confirmed without further cadaveric test data. This is particularly the case for the long duration impacts, where the current time-dependent injury criterion was established from a limited amount of experimental work and different impact types compared to head-first foam pit entries.

## Abbreviation

ATD: Anthropomorphic test device
